# Cannabis linked to improved sleep quality: A preliminary study

**DOI:** 10.1192/j.eurpsy.2021.1505

**Published:** 2021-08-13

**Authors:** J. Cebrian, G. Gonzalez-Cuevas

**Affiliations:** 1 Psychology, European University of Madrid, Madrid, Spain; 2 Biomedical And Pharmaceutical Sciences, Idaho State University, Meridian, United States of America

**Keywords:** Marijuana, Cannabis, sleep quality, PSQI

## Abstract

**Introduction:**

Sleep disorders are a substantial public health issue with serious consequences on patients’ quality of life. Cannabis has been recently suggested as a potential treatment for patients with sleep disorders; however, research on the relationship between cannabis and sleep is still in its infancy.

**Objectives:**

The aim of this investigation was to assess whether cannabis use was associated with improved sleep quality.

**Methods:**

Our study comprised 173 participants, 42 cannabis users and 131 non-cannabis users, who completed the Pittsburgh Sleep Quality Index (PSQI), the most common self-reported measure of sleep quality. The scale provides a global PSQI score and seven component domain scores, including subjective sleep quality, sleep latency, sleep duration, habitual sleep efficiency, sleep disturbances, use of sleep medication, and daytime functions.

**Results:**

Cannabis users self-reported statistically significantly healthier scores than non-cannabis users in the global PSQI as well as the specific domains of subjective sleep quality, sleep latency, as well as sleep disturbances.

**Conclusions:**

This preliminary evidence points to the possibility that cannabis could provide effective treatment for patients with sleep disorders. Research into the constituents of cannabis that may have a differential impact on sleep and sleep disorders is warranted.

